# Raised levels of chemerin in women with preeclampsia: A meta-analysis

**DOI:** 10.17305/bb.2023.9671

**Published:** 2024-06-01

**Authors:** Yue Xie, Xiaozhen Quan, Xuezhou Yang

**Affiliations:** 1 Department of Reproductive Center, Xiangyang Central Hospital, Affiliated Hospital of Hubei University of Arts and Science, Xiangyang City, Hubei Province, China

**Keywords:** Chemerin, adipokine, preeclampsia (PE), biomarker, meta-analysis

## Abstract

Chemerin is a multifunctional adipokine associated with systemic inflammation, angiogenesis, and oxidative stress. Emerging evidence suggests a potential link between chemerin and the pathogenesis of preeclampsia (PE). In this systematic review and meta-analysis, we aimed to evaluate the serum chemerin levels in women with PE. A systematic search was conducted across Medline, Web of Science, and Embase databases from inception until April 15, 2023, to identify studies comparing serum chemerin levels in pregnant women with and without PE. A random-effects model was employed to pool the results, accounting for heterogeneity. Thirteen datasets from ten observational studies, encompassing 832 women with PE and 1298 healthy pregnant women, were analyzed. The pooled findings indicated a statistically significant elevation in serum chemerin levels in women with PE compared to controls (mean difference [MD] ═ 89.56 ng/mL, 95% confidence interval [CI] 62.14–116.98; *P* < 0.001; *I*^2^ ═ 87%). The subgroup analysis revealed consistent findings across studies that measured chemerin levels before or after the diagnosis of PE, studies that did or did not match the body mass index (BMI), and studies with varying quality scores (*P* values for subgroup differences were all > 0.05). Compared to controls, women with severe PE exhibited a significantly greater increase in serum chemerin levels than those with mild PE (*P* value for subgroup difference ═ 0.007). Additionally, meta-regression analysis results suggested that the mean BMI of the included pregnant women might positively modify the difference in circulating chemerin levels between women with and without PE (coefficient ═ 8.92; *P* ═ 0.045). In conclusion, this meta-analysis suggests a positive correlation between elevated serum chemerin levels and PE diagnosis in comparison to pregnant women without the condition.

## Introduction

Preeclampsia (PE) is a pregnancy-related complication characterized by elevated blood pressure and proteinuria, emerging after 20 weeks of gestation [1, 2]. The incidence of PE among pregnant women is reported to range from 3% to 8% in previous studies [3]. Clinically, PE is associated with a spectrum of unfavorable maternal and perinatal outcomes [4]. Maternal complications of PE include renal injury, neurological impairment, and eclamptic seizures during pregnancy, as well as preterm birth and low birth weight [5]. Moreover, women with a history of PE are more likely to experience adverse cardiovascular events in the future [6, 7]. Notwithstanding, the most efficient approach to managing PE involves inducing iatrogenic preterm birth through premature delivery of the fetus [8]. Accordingly, ongoing endeavors are being undertaken to comprehend the fundamental mechanisms underlying the pathogenesis of PE, aiming to identify promising strategies for timely diagnosis and efficient treatment of the condition.

Chemerin, an immunomodulatory adipokine, was initially identified as a gene for the retinoic acid-receptor responder 2 (RARRES2) in lesions of psoriatic skin [9, 10]. Further investigations have revealed its involvement in various other biological processes, such as systemic inflammation, angiogenesis, and oxidative stress [11, 12], all of which are suggested to contribute to the development of PE [13]. According to a growing body of evidence, changes in serum chemerin levels can be detected in individuals with hypertension, coronary artery disease, chronic kidney disease, and cancer [14–17]. However, there exists a dearth of thorough assessment regarding the correlation between chemerin and PE [18]. Consequently, we conducted a systematic review and menmta-analysis to appraise the magnitude of serum chemerin in females afflicted with PE.

## Materials and methods

The present investigation adhered to the guidelines of the Preferred Reporting Items for Systematic Reviews and Meta-Analyses (PRISMA) 2020 [19, 20] and the Cochrane Handbook for Systematic Reviews of Interventions [21]. The protocol for this systematic review and meta-analysis has been registered on the International Platform of Registered Systematic Review and Meta-analysis Protocols (INPLASY, https://inplasy.com/register/) with the registration code of INPLASY202360068.

### Literature search

The present study utilized a comprehensive search strategy to obtain relevant literature from Medline, Web of Science, and Embase. The search strategy was defined as “chemerin” and “preeclampsia” or “pre-eclampsia” or “eclampsia” or “pregnancy-induced hypertension” or “PIH” or “toxemia” or “edema-proteinuria-hypertension gestosis” or “EPH”. Only studies published in English and involving human subjects were included. Additionally, the citations of related original and review articles were manually screened to supplement the search process. The literature searches were conducted up to April 15, 2023. The EndNote X9 software (Clarivate Analytics, Philadelphia, PA, USA) was used for management, utilizing its automatic duplicate checking function to locate and eliminate duplicate documents.

### Selection of studies

The present study employed specific inclusion criteria, which encompassed: (1) observational studies published as full-length articles in peer-reviewed journals; (2) inclusion of women diagnosed with PE as cases, and healthy pregnant women without PE as controls; (3) measurement and comparison of circulating chemerin levels between the two groups; and (4) reporting of the difference in the circulating concentrations of chemerin in means with standard deviations (SDs), or availability for calculation of such values. The diagnosis of PE was based on the criteria outlined in the included studies. Exclusion criteria for the present study included preclinical investigations, review articles, studies lacking cases of PE, and studies failing to report the blood chemerin levels between cases and controls. In the event of encountering studies with overlapping patient populations, the study with the largest sample size was included.

### Data collection and study quality assessment

The data was obtained through a collaborative effort by two independent authors who conducted a thorough search and evaluation process. In instances of disagreement, discussions were held with the corresponding author to reach a resolution. The collected data encompassed various aspects, including the author, year, and location of the study, participant characteristics, such as the number of cases and controls and their mean ages, diagnostic criteria for PE, source and characteristics of controls, timing of blood sampling, methods for measuring serum chemerin, and variables matched or controlled between cases and controls. The Newcastle–Ottawa Scale (NOS) was utilized to evaluate the quality of the studies [22]. The NOS assesses studies based on three overarching criteria: selection of cases and controls, comparability between groups, and exposure measurement. The total score ranges from 1 to 9, with a higher score indicating superior study quality.

### Ethical statement

Ethical approval and written informed consent for participation were not required for this study in accordance with local/national guidelines.

### Statistical analysis

The units of ng/mL, µg/L, and pg/mL were used among the included studies when reporting the levels of chemerin, and these were converted to ng/mL for consistency (1 µg/L ═ 1 ng/mL; 1 pg/mL ═ 0.001 ng/mL). Since all of the included studies measured chemerin using the enzyme-linked immunosorbent assay (ELISA), the mean difference (MD) and corresponding 95% confidence interval (95% CI) were utilized to present the variance in serum levels of chemerin between pregnant women with and without PE [23], rather than using the standardized MD. To assess the degree of heterogeneity between studies, the Cochrane *Q* test was conducted and the *I*^2^ statistic was estimated [23, 24]. An *I*^2^ value exceeding 50% indicates heterogeneity. A random-effects model was employed to pool the results, accounting for potential between-study heterogeneity [21]. To evaluate the robustness of the findings, sensitivity analyses were performed by sequentially removing individual datasets. Additionally, subgroup analyses were conducted to investigate the impact of various study characteristics, including the timing of blood sampling (pre- or post-diagnosis of PE), body mass index (BMI) matching between women with and without PE, the severity of PE, and study quality scores, on the results. Women diagnosed with PE who exhibited any of the following risk factors, including but not limited to headache, visual or cerebral disturbance, elevated liver enzymes, thrombocytopenia, dyspnea due to pulmonary edema, progressive renal failure, systolic blood pressure ≥ 160 mmHg and/or diastolic blood pressure ≥ 110 mmHg, were classified as having severe PE. Conversely, those who did not exhibit any of the aforementioned risk factors were classified as having mild PE [25]. In addition, meta-regression was performed to evaluate the potential influence of study characteristics on the outcome in continuous variables, which included mean BMI, mean age, and sample size of the study [21]. To ascertain the presence of publication bias, the construction of funnel plots and their visual inspection for symmetry were employed, as well as the implementation of Egger’s regression analysis [26]. The statistical analysis was carried out using RevMan (Version 5.1; Cochrane Collaboration, Oxford, UK) and Stata software (version 12.0; Stata Corporation, College Station, TX, USA), with statistical significance indicated by *P* values < 0.05.

**Figure 1.**
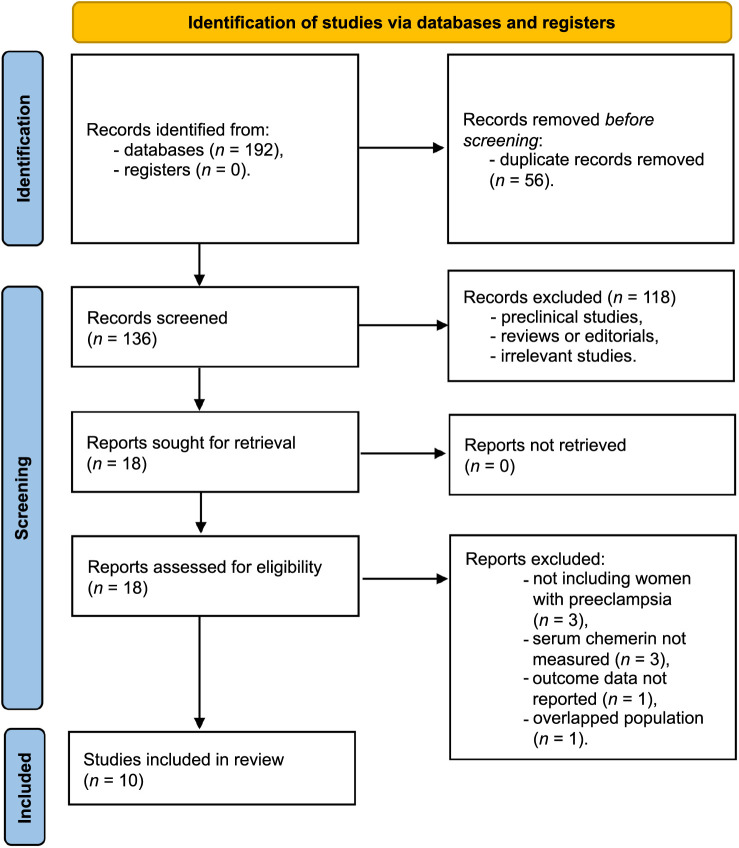
**A flowchart depicting the methodology of database exploration and study identification.** Out of the 192 initially identified studies, duplicates were first excluded, followed by the removal of articles non-compliant with the meta-analysis criteria. Upon independent review of the remaining articles by the authors, further exclusions were made for reasons listed in the figure, resulting in a final inclusion of ten observational studies in the meta-analysis.

## Results

### Study retrieval

Figure 1 illustrates that a search of electronic databases yielded 192 articles, of which 136 remained after duplicates were eliminated. Out of these 136 titles and abstracts screened for the meta-analysis, 118 were excluded due to non-compliance with the meta-analysis criteria. Upon independent reading of the full texts by two authors, eight of the remaining 18 studies were excluded for reasons listed in Figure 1. Consequently, the meta-analysis included ten observational studies [27–36].

**Figure 2.**
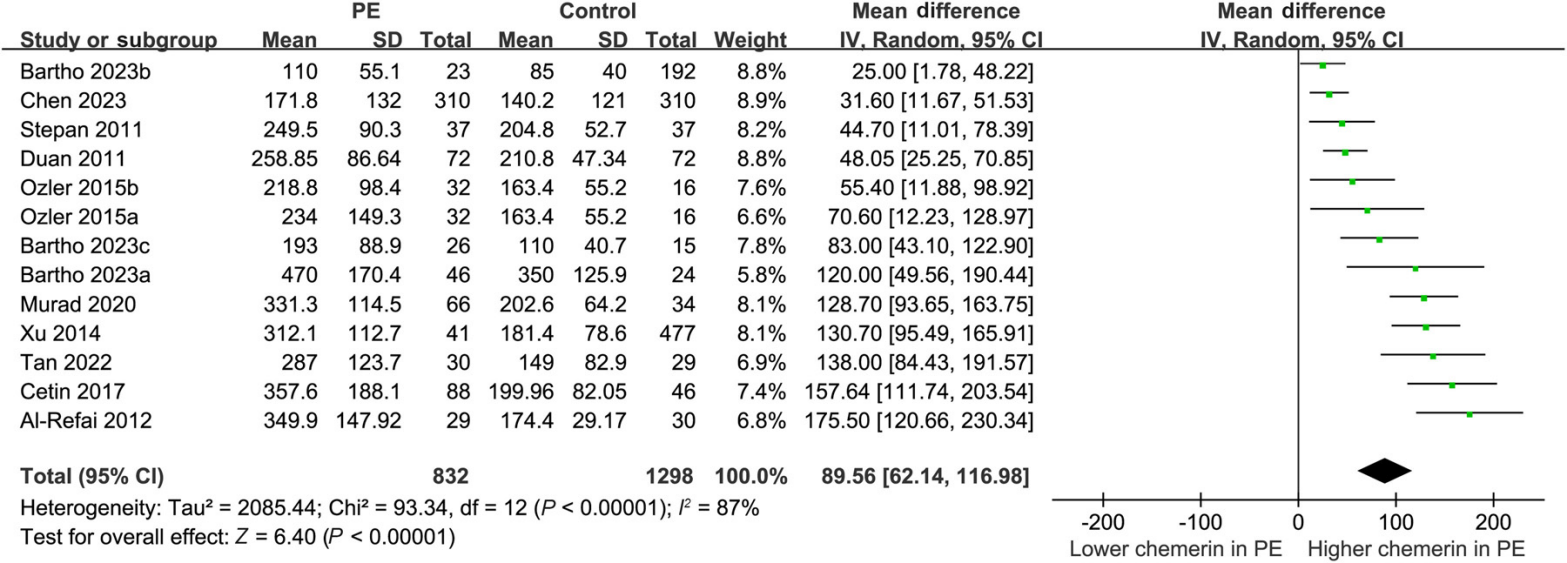
**Forest plots depicting the meta-analysis of alterations in serum chemerin levels among women diagnosed with PE.** PE: Preeclampsia; CI: Confidence interval; SD: Standard deviation.

**Table 1:** Characteristics of the included studies

**Study**	**Country**	**Study design**	**Maternal age (years)**	**Diagnosis of PE**	**Mean BMI (kg/m^2^)**	**Source of control**	**No. of PE**	**No. of control**	**Timing of sampling**	**Methods and units used for measuring chemerin**	**Variables matched or adjusted**
Duan, 2011	China	C-C	29	ACOG 2002	21	Healthy pregnant women	72	72	Third trimester	ELISA (ng/mL)	Age and GA
Stepan, 2011	Germany	C-C	30.5	ACOG 2002	22.2	Healthy pregnant women	37	37	Second or third trimester	ELISA (µg/L)	Age, BMI, and GA
Al-Refai, 2012	Egypt	C-C	33.2	ACOG 2002	29.8	Healthy pregnant women	29	30	Third trimester	ELISA (ng/mL)	Age, BMI, and GA
Xu, 2014	China	Prospective	25.2	ACOG 2002	23.1	Normotensive pregnant women	41	477	First trimester, before the diagnosis of PE	ELISA (ng/mL)	Age and GA
Ozler, 2015a	Turkey	C-C	31.2	ACOG 2002	28.2	Healthy pregnant women	32	16	Second or third trimester	ELISA (µg/L)	Age, BMI, and GA
Ozler, 2015b	Turkey	C-C	31.2	ACOG 2002	24.5	Healthy pregnant women	32	16	Second or third trimester	ELISA (µg/L)	Age and GA
Cetin, 2017	Turkey	C-C	27.8	ACOG 2002	26.2	Healthy pregnant women	88	46	Second or third trimester	ELISA (ng/mL)	Age and GA
Murad, 2020	Iraq	C-C	25.3	ACOG 2002	28.1	Healthy pregnant women	66	34	Second or third trimester	ELISA (ng/mL)	Age, BMI, and GA
Tan, 2022	The Netherlands	C-C	32.5	ISSHP 2001	25	Healthy pregnant women	30	29	Third trimester	ELISA (ng/mL)	Age, BMI, and GA
Bartho, 2023a	Australia	Prospective	31.6	ACOG 2013	26.2	Healthy pregnant women	46	24	Second or third trimester, before the diagnosis of PE	ELISA (pg/mL)	Age
Bartho, 2023b	Australia	Prospective	32	ACOG 2013	27.5	Healthy pregnant women	23	182	Third trimester, before the diagnosis of PE	ELISA (pg/mL)	Age and GA
Bartho, 2023c	South Africa	C-C	31.8	ACOG 2013	26.1	Healthy pregnant women	26	15	Third trimester	ELISA (pg/mL)	Age, BMI, and GA
Chen, 2023	China	C-C	33.3	ACOG 2013	22.7	Healthy pregnant women	310	310	Third trimester	ELISA (ng/mL)	Age, BMI, and GA

In the study conducted by Ozler et al., two distinct cohorts were formed based on the obesity status of the women with PE. In the table, these cohorts are denoted by the letters “a” and “b” next to the author’s name, which appears twice. Similarly, the study by Bartho et al. reported results from three independent cohorts. In the meta-analysis, datasets from these cohorts were included separately. On the table, these cohorts are denoted by the letters “a,” “b,” and “c” next to the author’s name, indicating the three different cohorts. Overall, 13 cohorts from ten observational studies were included. BMI: Body mass index; PE: Preeclampsia; C-C: Case-control; ACOG: American College of Obstetricians and Gynecologists; ISSHP: The International Society for the Study of Hypertension in Pregnancy; ELISA: Enzyme-linked immunosorbent assay; GA: Gestational age.

### Study characteristics

An overview of the study characteristics is provided in Table 1. Since one of the included studies categorized two cohorts based on the obesity status of the included women with PE [31], and another study reported results of three independent cohorts [35], datasets from these sources were included independently in the meta-analysis. Overall, 13 cohorts from the ten observational studies were included. Ten of these were designed as case-control studies [27–29, 31–36], while the other three were of a prospective design [30, 35]. These studies were conducted in China, Germany, Egypt, Turkey, Iraq, the Netherlands, Australia, and South Africa, and were published between 2011 and 2023. The study comprised 832 women diagnosed with PE and 1298 healthy pregnant women, with mean ages ranging from 25.3 to 33.3 years. The diagnostic criteria for PE were aligned with the guidelines set forth by the American College of Obstetricians and Gynecologists (ACOG) in 2002 in seven studies [27–33], with the ACOG 2013 guideline in two studies [35, 36], and with the criteria established by the International Society for the Study of Hypertension in Pregnancy (ISSHP) in another study [34]. Blood sampling was conducted before the diagnosis of PE in three cohorts [30, 35], and after the diagnosis of PE in the other ten cohorts [27–29, 31–36]. The ELISA was used to measure serum chemerin in all of the included studies. Maternal age was matched between women with and without PE in all of the included cohorts, while gestational age (GA) and BMI were matched in 12 [27–36] and 7 cohorts [28, 29, 31, 33–36], respectively. Overall, the studies incorporated in the analysis were awarded a score of seven or eight stars on the NOS, signifying a high level of study quality (Table 2).

**Table 2:** Study quality evaluation via the Newcastle–Ottawa Scale

	**Adequate definition of the cases**	**Representativeness of the cases**	**Selection of controls**	**Definition of controls**	**Controlled for GA**	**Controlled for BMI**	**Ascertainment of the exposure**	**Same method of ascertainment of exposure for cases and controls**	**Non-response rate**	**Overall**
Duan, 2011	1	0	1	1	1	0	1	1	1	7
Stepan, 2011	1	0	1	1	1	1	1	1	1	8
Al-Refai, 2012	1	0	1	1	1	1	1	1	1	8
Xu, 2014	1	1	1	1	1	0	1	1	1	8
Ozler, 2015a	1	0	1	1	1	1	1	1	1	8
Ozler, 2015b	1	0	1	1	1	0	1	1	1	7
Cetin, 2017	1	0	1	1	1	0	1	1	1	7
Murad, 2020	1	0	1	1	1	1	1	1	1	8
Tan, 2022	1	0	1	1	1	1	1	1	1	8
Bartho, 2023a	1	1	1	1	0	0	1	1	1	7
Bartho, 2023b	1	1	1	1	1	0	1	1	1	8
Bartho, 2023c	1	0	1	1	1	1	1	1	1	8
Chen, 2023	1	0	1	1	1	1	1	1	1	8

In the study conducted by Ozler et al., two distinct cohorts were formed based on the obesity status of the women with PE. In the table, these cohorts are denoted by the letters “a” and “b” next to the author’s name, which appears twice. Similarly, the study by Bartho et al. reported results from three independent cohorts. In the meta-analysis, datasets from these cohorts were included separately. On the table, these cohorts are denoted by the letters “a,” “b,” and “c” next to the author’s name, indicating the three different cohorts. The Newcastle–Ottawa Scale was utilized to evaluate the quality of the studies. The total score ranges from 1 to 9, with a higher score indicating superior study quality. Overall, the studies incorporated in the analysis were awarded a score of seven or eight stars on the Newcastle–Ottawa Scale, signifying a high level of study quality. GA: Gestational age; BMI: Body mass index.

### Results of the meta-analysis

**Figure 3.**
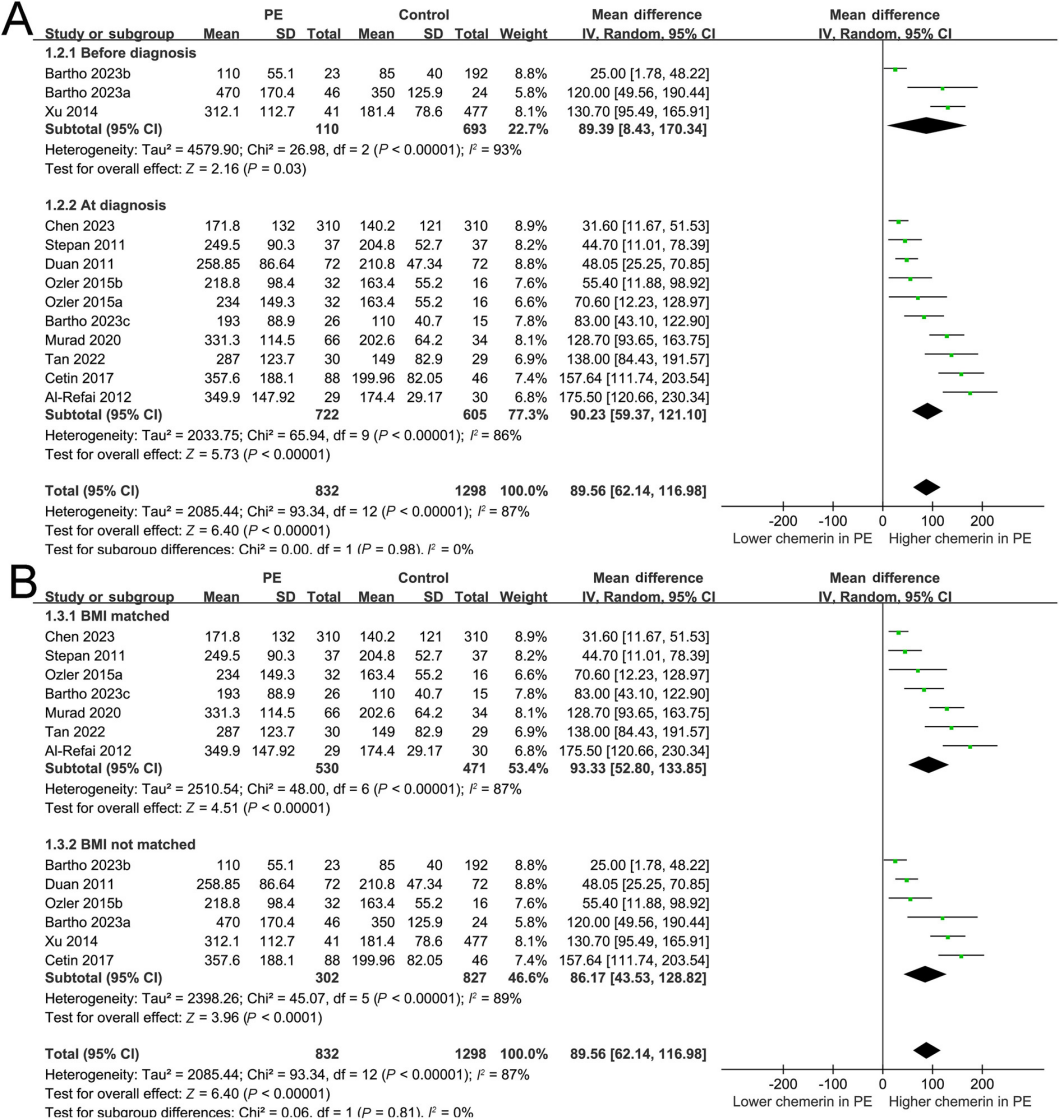
**Forest plots depicting the subgroup analysis of alterations in serum chemerin levels among women diagnosed with PE.** (A) Subgroup analysis conducted based on the timing of blood sampling (chemerin levels measurement prior to or following the diagnosis of PE); (B) Subgroup analysis conducted based on whether the BMI was matched or not between women with and without PE. PE: Preeclampsia; BMI: Body mass index; SD: Standard deviation; CI: Confidence interval.

**Figure 4.**
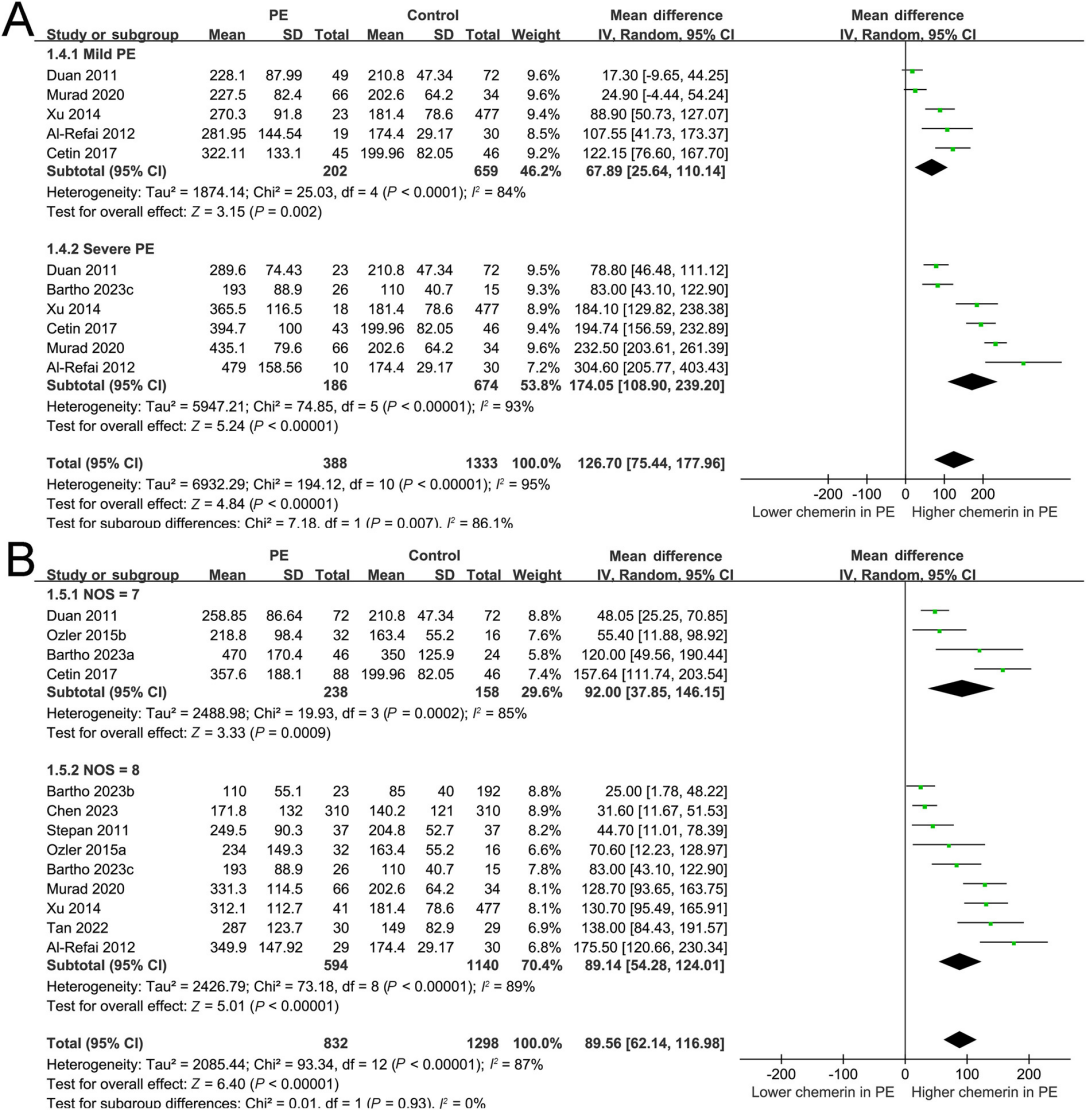
**Forest plots depicting the subgroup analysis of alterations in serum chemerin levels among women diagnosed with PE.** (A) Subgroup analysis conducted based on the severity of PE; (B) Subgroup analysis conducted based on the quality score of the included study. PE: Preeclampsia; SD: Standard deviation; CI: Confidence interval; NOS: Newcastle–Ottawa Scale.

**Figure 5.**
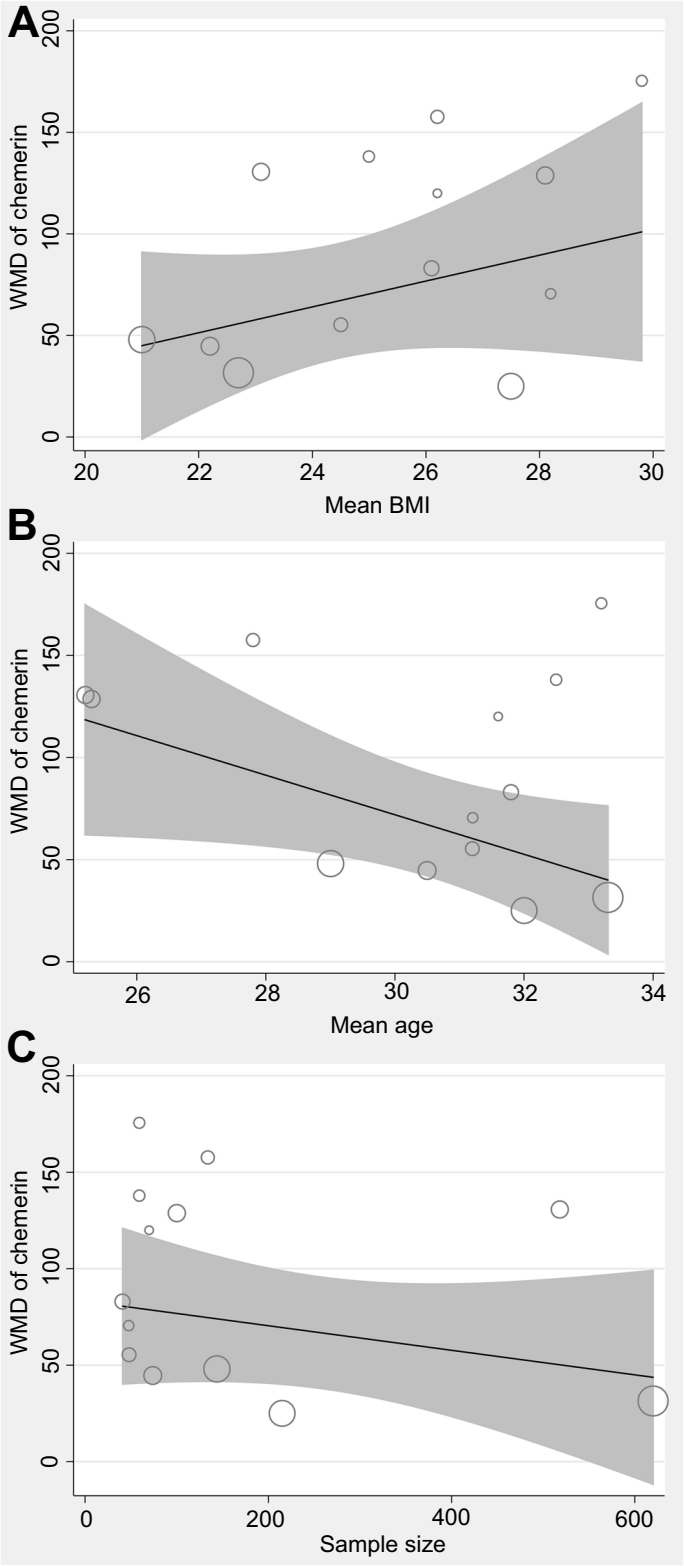
**Plots for the meta-regression analysis evaluating the influence of mean BMI, age, and sample size on the association between chemerin and PE.** (A) Meta-regression analysis conducted to evaluate the influence of mean BMI on the difference in chemerin levels between women with and without PE; (B) Meta-regression analysis conducted to evaluate the influence of mean age on the difference in chemerin levels between women with and without PE; (C) Meta-regression analysis conducted to evaluate the influence of sample size on the difference in chemerin levels between women with and without PE. BMI: Body mass index; PE: Preeclampsia; WMD: Weighted mean difference.

The utilization of a random-effects model to pool results indicated that women diagnosed with PE exhibited a statistically significant elevation in serum chemerin concentration when compared to control subjects (MD ═ 89.56 ng/mL, 95% CI 62.14–116.98 ng/mL; *P* < 0.001; *I*^2^ ═ 87%; Figure 2). The consistent results of the sensitivity analysis, which involved the exclusion of one dataset at a time, were demonstrated through a range of MD from 82.87 to 95.80 ng/mL (*P* < 0.05; Table 3). Moreover, none of the included studies seemed to significantly contribute to the heterogeneity of the meta-analysis (residual *I*^2^: 86%–88%; Table 3). Furthermore, when the sensitivity analysis was limited to cohorts with PE diagnosed by the ACOG criteria, the results remained consistent (MD ═ 85.86 ng/mL, 95% CI 57.89–113.83; *P* < 0.001; *I*^2^ ═ 87%). The subgroup analysis demonstrated consistent findings across studies that measured chemerin levels prior to or following the diagnosis of PE (*P* value for subgroup analysis ═ 0.98; Figure 3A), as well as in studies that did or did not match BMI between cases and controls (*P* value for subgroup analysis ═ 0.81; Figure 3B). Interestingly, women with severe PE (MD ═ 174.05 ng/mL, 95% CI 108.90–239.20; *P* < 0.001) were associated with a more remarkable increment of serum chemerin as compared to those with mild PE (MD ═ 67.89 ng/mL, 95% CI 25.64–110.14; *P* ═ 0.002; *P* value for subgroup difference ═ 0.007; Figure 4A). Subsequent to the subgroup analysis, it was observed that studies of varying quality scores yielded consistent outcomes (*P* value for subgroup difference ═ 0.93; Figure 4B). Further meta-regression analysis suggested that the mean BMI of the included pregnant women may positively modify the difference in circulating chemerin levels between women with and without PE (coefficient ═ 8.92, 95% CI 0.51–17.33; *P* ═ 0.045; Figure 5A), but not for other variables such as mean age (coefficient ═ −6.25, 95% CI −17.77 to 5.28; *P* ═ 0.258; Figure 5B) or the sample size of the included study (coefficient ═ −0.058, 95% CI −0.231 to 0.114; *P* ═ 0.473; Figure 5C).

**Table 3:** Results of the sensitivity analysis involving the exclusion of one dataset at a time, and the assessment of contribution to heterogeneity

**Dataset excluded**	**MD (95% CI) (ng/mL)**	***P* for effect**	***I* ^2^**
Duan, 2011	94.00 (63.16 – 124.85)	<0.001	88%
Stepan, 2011	93.82 (64.16 – 123.49)	<0.001	88%
Al-Refai, 2012	82.87 (56.29 – 109.45)	<0.001	86%
Xu, 2014	85.66 (57.98 – 113.34)	<0.001	86%
Ozler, 2015a	91.04 (62.13 – 119.95)	<0.001	88%
Ozler, 2015b	92.57 (63.24 – 121.91)	<0.001	88%
Cetin, 2017	83.66 (56.84 – 110.48)	<0.001	86%
Murad, 2020	85.87 (58.06 – 113.67)	<0.001	86%
Tan, 2022	85.86 (57.89 – 113.83)	<0.001	87%
Bartho, 2023a	87.71 (59.37 – 116.04)	<0.001	88%
Bartho, 2023b	95.80 (67.02 – 124.58)	<0.001	86%
Bartho, 2023c	90.34 (60.89 – 119.78)	<0.001	88%
Chen, 2023	95.38 (66.01 – 124.75)	<0.001	86%

In the study conducted by Ozler et al., two distinct cohorts were formed based on the obesity status of the women with PE. In the table, these cohorts are denoted by the letters “a” and “b” next to the author’s name, which appears twice. Similarly, the study by Bartho et al. reported results from three independent cohorts. In the meta-analysis, datasets from these cohorts were included separately. On the table, these cohorts are denoted by the letters “a,” “b,” and “c” next to the author’s name, indicating the three different cohorts. MD: Mean difference; CI: Confidence interval.

### Publication bias

**Figure 6.**
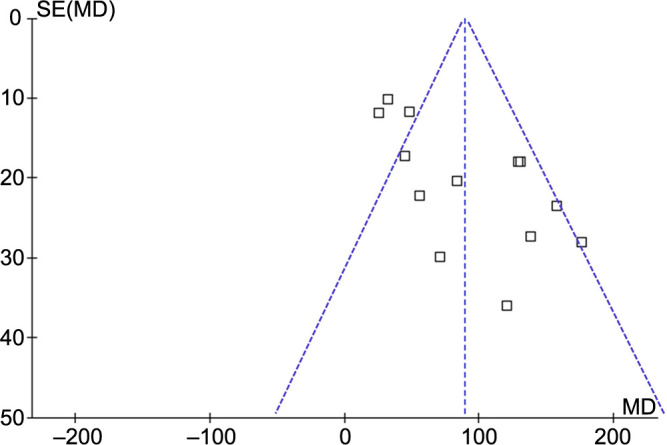
**Funnel plots utilized to assess potential publication bias in the meta-analysis examining alterations in serum chemerin levels among women diagnosed with PE.** PE: Preeclampsia; SE: Standard error; MD: Mean difference.

Figure 6 displays the funnel plots utilized in the meta-analysis to compare serum chemerin levels in women with and without PE. The symmetry of the plot suggests a low risk of publication bias. Additionally, the results of Egger’s regression tests do not indicate any significant publication bias (*P* ═ 0.31).

## Discussion

The present meta-analysis synthesized data from ten observational studies, comprising 13 datasets, to investigate the association between serum chemerin levels and PE in women. The findings revealed that women with PE exhibited significantly elevated serum chemerin levels compared to healthy pregnant controls. The robustness of these results was affirmed through sensitivity analysis, which involved the exclusion of individual datasets. Furthermore, subgroup analyses revealed that the heightened serum concentration of chemerin in women with PE was detectable both before and after the diagnosis of PE, across studies with and without BMI matching between cases and controls, and across studies with varying quality scores. Notably, a more pronounced elevation of serum chemerin was observed in women with severe PE compared to those with mild PE. Moreover, further meta-regression analysis suggested that the mean BMI of the included pregnant women may positively modify the difference in circulating chemerin between women with and without PE, which could be a significant contributor to heterogeneity. Collectively, our meta-analysis provides evidence that women with PE exhibit a significantly elevated serum concentration of chemerin compared to healthy pregnant controls.

To date, there has not been a comprehensive meta-analysis examining the correlation between serum chemerin levels and PE. The results of this meta-analysis indicate a significant elevation in serum chemerin levels among women with PE when compared to their healthy pregnant counterparts. This suggests that alterations in chemerin may contribute to the pathogenesis of PE. Our subgroup analysis findings indicate that pregnant women with higher serum concentrations of chemerin may be at an increased risk for developing PE, both pre- and post-diagnosis. These results hint at the potential of serum chemerin as a biomarker for PE development. Further large-scale prospective studies are required to validate these findings. Moreover, our study revealed that the correlation between serum chemerin and PE was not confounded by obesity, as higher serum chemerin levels were observed in studies where cases and controls were matched for BMI. This is significant as it has been shown that obesity leads to an elevation in the systemic concentration of chemerin, which functions as an adipokine [37]. Furthermore, our study revealed a more notable elevation in serum chemerin concentration among women diagnosed with severe PE as compared to those with mild PE, indicating a plausible association between the systemic level of chemerin and the severity of PE. Finally, meta-regression suggests that the mean BMI of the included pregnant women may positively modify the difference in circulating chemerin between women with and without PE, indicating that the potential difference in circulating chemerin between PE and normal pregnancy may be more remarkable in women with higher BMI. This finding is of great significance as chemerin, being an adipokine, may be influenced by an individual’s obesity status [38].

Currently, the precise mechanisms underlying the correlation between chemerin and PE have yet to be fully elucidated. A previous preclinical investigation demonstrated that chemerin upregulation was evident in both in vivo and in vitro models of PE, facilitating the pyroptosis and inflammation of trophoblasts, thereby contributing to the development of PE [39]. A subsequent study posited that chemerin, by activating the chemerin chemokine-like receptor 1 (CMKLR1)/protein kinase B (Akt)/CCAAT enhancer binding protein alpha (CEBPα) axis, establishes a positive feedback loop that fosters M1 macrophage polarization, and hinders trophoblast migration/invasion and angiogenesis, ultimately playing a role in the development of PE [40]. A recent study employed lentivirus-mediated trophoblast-specific gene manipulation to induce chemerin overexpression [34]. The study revealed that upregulation of placental chemerin synthesis disrupts normal placental development through its CMKLR1 receptor, ultimately leading to fetal growth restriction/resorption and the onset of PE [34]. These preclinical findings, in conjunction with the results of the present meta-analysis, suggest that chemerin may serve as a promising biomarker for PE.

The meta-analysis has several methodological strengths. Specifically, our exhaustive search across three electronic databases yielded ten pertinent observational studies that aligned with our objective. Additionally, the included studies strived to mitigate potential confounding factors, such as maternal age, GA, and BMI, through matching or control procedures between case and control groups, aiming to minimize the possible influences of such factors on the serum chemerin concentrations. Lastly, the consistency of results from the sensitivity analyses and multiple predefined subgroup analyses further reinforces the robustness and stability of the findings.

Our study also bears certain limitations. Firstly, the number of studies and patients included in the analysis is limited, thus underscoring the need for validation of the results through large-scale research endeavors. Secondly, the majority of the studies included in the analysis were case-control studies, highlighting the need for large-scale prospective studies to ascertain whether a high serum chemerin level is independently associated with the development of PE. Moreover, given the meta-analytical nature of our study encompassing observational studies, it was not feasible to establish a definitive causal association between the rise in systemic chemerin and the onset of PE. Despite the efforts to match or control for various potential confounding variables among women with and without PE, residual factors that might affect the correlation between chemerin and PE may still prevail. Finally, various cytokines besides chemerin, such as inflammatory cytokines [41] and other adipokines like leptin [42], have been suggested to play roles in the pathogenesis of PE. At the current stage, we could not determine the interactions between chemerin and these cytokines in women with PE. Future studies are warranted for further investigation.

## Conclusion

In summary, the occurrence of PE in women is associated with a significant elevation in serum chemerin levels compared to healthy pregnant controls, evident even prior to the diagnosis of PE. Moreover, these observations appear to be independent of BMI, with the rise in serum chemerin levels potentially being more pronounced in women diagnosed with severe PE compared to those with mild PE. Collectively, these findings indicate that chemerin could serve as a novel biomarker for PE in pregnant women.

## Data Availability

All the data generated during the study are within the manuscript.
